# Comparative Effects of Three Iron Chelation Therapies on the Quality of Life of Greek Patients with Homozygous Transfusion-Dependent Beta-Thalassemia

**DOI:** 10.5402/2012/139862

**Published:** 2012-12-17

**Authors:** Vasilis Goulas, Alexandra Kourakli-Symeonidis, Charalambos Camoutsis

**Affiliations:** ^1^Department of Pharmacy, General Hospital of Agrinio, Kokkali Street, 30100 Agrinio, Greece; ^2^Thalassemia and Hemoglobinopathies Centre, Hematology Division, University Hospital of Patras, Patras, Greece; ^3^Laboratory of Pharmaceutical Chemistry, Department of Pharmacy, University of Patras, Patras, Greece

## Abstract

This prospective study assessed the quality of life of patients with homozygous transfusion-dependent beta-thalassemia in Greece receiving three different iron chelation treatments. Patients enrolled were receiving one of the following chelation therapies: deferoxamine (*n* = 21), deferasirox (*n* = 75), or deferoxamine in combination with deferiprone (*n* = 39). The three groups were compared in terms of their quality of life, satisfaction and adherence to treatment, control of their health, and self-esteem through the completion of five questionnaires. A higher percentage of patients receiving deferoxamine felt that their treatment negatively influenced their body and skin appearance and limited their ability to work, attend school, and perform daily tasks (*P* = 0.0066). The adherence to treatment rate and self-esteem were the lowest in the deferoxamine group (*P* < 0.05). The deferoxamine group also had the lowest physical component summary score in the SF-36 questionnaire (*P* = 0.014). This study suggests that the quality of life of beta-thalassemia patients receiving chelation therapy is dependent on the type of iron chelation treatment they receive. The study provides insight into important factors associated with the quality of life of these patients, which are essential for developing a more suitable clinical support team and counseling in order to maximize the treatment benefits for these patients in daily clinical practice.

## 1. Introduction

Beta-thalassemia is a genetically inherited disorder characterized by reduced synthesis of the beta-hemoglobin chain which in turn results in reduced synthesis of hemoglobin A (HbA). To date more than 1,000 mutations are known that influence the structure or synthesis of the alpha- and beta-globin chains that make up HbA and which are listed in the HbVar database (HbVar), a database of all the mutations related to thalassemia and the variations of hemoglobin [1, 2]. In Greece, in addition to common mutations with distribution patterns that vary between particular region(s) of the country [3], some rare mutations have been reported. All these mutations are included in the Hellenic National Genetic database, a database that lists mutations of all inherited disorders present in the Greek population [4].

Treatment of patients with thalassemia major consists of regular blood transfusions and iron chelation therapy, which is vital to prevent excess iron buildup in the body. In Europe there are three iron chelating agents available: deferoxamine (DFO, Desferal), an iron chelator given by infusion, and two oral chelators deferiprone (DFP, Ferriprox) and deferasirox (DFX, Exjade). Treatment with iron chelators has significantly increased the life expectancy of affected individuals into the third to fifth decade [5], while simultaneously decreasing the comorbidities of the disease [6].

Despite advancements in care, patients with transfusion-dependent beta-thalassemia still present complications and often suffer from psychological problems due to their lifestyle [7–9]. While the effectiveness of iron chelation therapies has been thoroughly investigated, there is limited comparative information about the benefits of the therapies on the quality of life and self-esteem of the patients. Furthermore, while Greece is a country with high prevalence of beta-thalassemia, the quality of life of patients presenting with this disease and the effect of the type of iron chelation treatment on the patient's quality of life have not been evaluated. Thus, the objective of the present study was to compare the quality of life, self-esteem, and satisfaction and adherence to treatment of patients with homozygous transfusion-dependent beta-thalassemia in the Greek population receiving three different chelation treatments and to identify parameters affecting their quality of life. The SF-36 questionnaire was used in order to evaluate the quality of life in the 135 patients of the study. Three other questionnaires were administered which provided important information on factors varying among patients receiving different types of iron chelation therapy.

## 2. Methods

### 2.1. Patients

A total of 135 adults with transfusion-dependent homozygous beta-thalassemia attending the Thalassemia Units of the University Hospital of Rio-Patras and of the General Hospital of Agrinio between May 2009 and May 2011 were enrolled in the study. During this time period, 174 homozygous beta-thalassemia patients were attending the aforementioned two units; 39 did not return their questionnaires and did not provide reasons for dropping out of the study. Diagnosis with homozygous transfusion-dependent beta-thalassemia was the only criterion used for inclusion in the study. There were no exclusion criteria. The scientific committee of the study and the local ethics committees of the participating hospitals approved the study. All patients provided written informed consent for their participation. 

### 2.2. Evaluation Questionnaires

All patients were asked to answer the following four questionnaires: (i) the SF-36 questionnaire [10]; (ii) a Wallston's health locus of control scale, in which the patients answered 18 questions using a 6-point Likert-type scale response format [11] with 1 and 2 = strongly and moderately disagree, respectively, 3 = neither agree nor disagree, and 4, 5, and 6 = slightly, moderately, and strongly agree, respectively; (iii) a self-esteem questionnaire with 13 questions using a 5-point Likert-type scale (with 1 = very well and 5 = very poorly); (iv) a questionnaire about patient satisfaction from their current therapeutic chelator with 41 questions with responses varying from never to always. In addition, personal data and the hematological profile of the patients were obtained in order to be able to better evaluate the quality of life of the patients enrolled in our study. 

### 2.3. Statistical Analysis

All continuous variables are expressed as the mean ± standard deviation (SD). The categorical (nominal) variables are expressed as percentages of the total population. Comparisons of the categorical variables between the three therapies were performed by the chi square test or the Fisher test as appropriate. Differences in the continuous variables between the three groups were assessed by the Kruskal Wallis test. Differences were considered significant when the *P* value was <0.05. 

In order to investigate if chelation treatment is associated with patients' quality of life, univariate regression analysis was performed in which the eight scales and the two components of the SF-36 were set as dependent variables and chelation treatment was set as the independent variable. Multiple regression analysis was also performed for the two component scores (PCS, MCS) separately in order to evaluate their relationship with clinical and anthropometric characteristics. Specifically, gender, marital status, professional status, physical activity, smoking habits, years since diagnosis, years since chelation treatment onset, and other comorbidities were set as independent variables along with chelation treatment in order to investigate factors associated with the quality of life. Statistical analyses were performed with SPSS version 20.0 for Windows (Cary, NC, USA). 

## 3. Results

### 3.1. Patient Characteristics

Of the 135 adult patients with homozygous transfusion-dependent beta-thalassemia that were recruited in this study, 59 were males and 76 were females. The patients were divided into three groups based on the therapy they were receiving: the first group was receiving deferoxamine (DFO; Desferal, Novartis), the second group deferasirox (DFX; Exjade, Novartis), and the third group deferoxamine + deferiprone (DFP; Ferriprox, Demo S.A.) combination therapy. The mean age of the patients was 37.3 ± 10.1 for the DFO group, 34.3 ± 7.4 for the DFX group, and 37.8 ± 8.3 for the DFO + DFP group. The differences among the groups were not statistically significant. The demographic characteristics of the patients are shown in Table 1. More than half of the patients (75/135) were receiving DFX, while 39 and 21 were receiving combination therapy and DFO alone, respectively. The majority of the patients receiving DFO (19/21) and DFO + DFP (33/39) were not involved in sports, while 34/70 of DFX patients were, a statistically significant difference (*P* < 0.0001) (Table 1). 

### 3.2. Disease Characteristics

The disease characteristics of patients of the three groups are presented in Table 2. There were no significant differences among the three groups between their age at the time of diagnosis and their age when DFO treatment began. When examining the comorbidities, the patients receiving DFO had significantly higher percentages of myocardial dysfunction (33.3%) and hepatic dysfunction (38.1%), while 71.4% had undergone splenectomy and 14.3% suffered from allergies (Table 3). 

### 3.3. Results from Questionnaires

The SF-36 questionnaire was used as a measurement of the quality of life of the patients. This questionnaire consists of eight scales (1) physical functioning, (2) role limitations because of physical health problems, (3) bodily pain, (4) general health perceptions, (5) vitality (energy/fatigue), (6) social functioning, (7) role limitations due to emotional problems, and (8) general mental health (psychological distress and psychological wellbeing). The scores were calculated for respondents completing 50% or more of the items within a scale. Higher scores represent better health. The type of chelation treatment was proven to be statistically significantly associated with physical functioning (*P* = 0.048), role limitations due to physical health problems (*P* = 0.021), bodily pain (*P* = 0.015), vitality (*P* < 0.001), and mental health (*P* = 0.001) (Table 4). Pairwise comparisons performed in the aforementioned scales in order to ascertain differences among the treatments revealed that those who received DFX or DFO + DFP demonstrated significantly higher mean scores (better quality of life) than patients who received DFO alone, in all scales tested, apart from the bodily pain scale. In the bodily pain scale, only treatment with DFX resulted in a significantly higher mean score than treatment with DFO alone.

**Figure 1:**
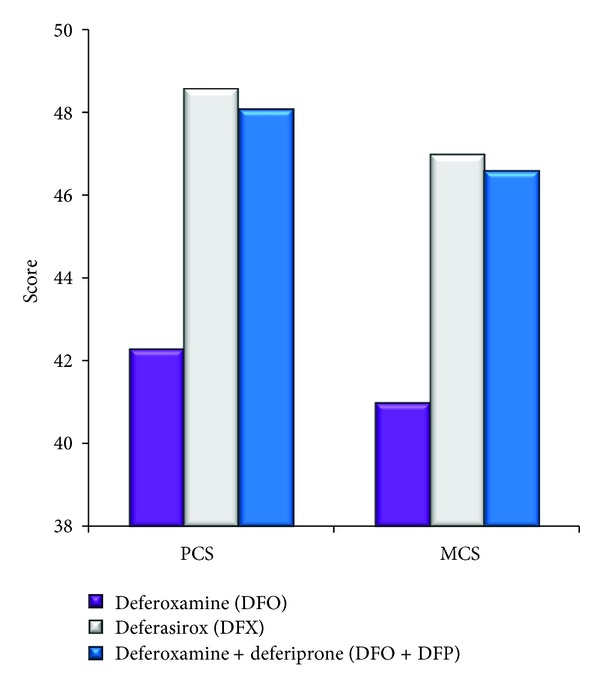
Quality of life for patients with the three different chelation treatments according to the SF-36 scale. The physical (PCS) and mental (MCS) component summary scores were calculated from data for 120 patients that had completed all parts of the questionnaire. These included 70 receiving DFX, 33 receiving DFO, and 17 receiving DFO + DFP. A statistically significant difference was noted between the chelation treatments for the PCS score (univariate analysis, *P* = 0.014). Pairwise comparisons demonstrated that both DFX and DFO + DFP were statistically different from DFO, but they were not different from each other. The MCS score did not differ among the chelation therapies (*P* = 0.08).

 For patients with available scores for all 8 scales, the physical health component score (PCS) and the mental health component score (MCS) were derived [10]. Univariate regression analysis demonstrated that the type of chelation treatment was statistically significantly associated with the PCS (Figure 1). In addition, pairwise comparisons revealed that patients who received DFX and combination treatment of DFO + DFP had a significantly higher mean PCS score (better physical health) than patients who received DFO. No statistically significant differences were observed between treatment with DFX and a combination treatment of DFO + DFP. 

The impact of the type of chelation therapy on the PCS was further confirmed by multivariate regression analysis (*P* = 0.001) in which anthropometric measures, specifically, gender, marital status, professional status, physical activity, smoking habits, years since diagnosis, years since chelation treatment onset, and other comorbidities were included. In this analysis, it also appeared that being married (*P* < 0.001) and a diagnosis of <30 years (*P* = 0.007) positively impacted the PCS score. Multivariate analysis with the MCS using the same anthropometric values listed above revealed that chelation treatment significantly impacted the MCS score (*P* = 0.009). 

In addition, patients completed Wallston's questionnaire pertaining to the control of their health. This questionnaire assesses five dimensions of control of one's health, “internal,” “chance,” “powerful others,” “doctors,” and “other people.” For each of these five dimensions, higher scores represent those that more strongly believe that they have little control over their health and that their health is dependent on the specific dimension examined [11]. According to the results of Wallston's 18-item Health Locus of Control Scale, patients only differed slightly (*P* = 0.0438) in their belief that their doctors had control of their personal health status. The highest score (13.4 ± 2.1) was observed in the DFO group of patients compared to the DFX (11.5 ± 3.0) and DFO + DFP (12.1 ± 3.0) groups, meaning that those were the ones that felt that their doctors had greatest control over their health. There were no statistically significant differences noted among the groups for the other four dimensions. 

The majority of patients felt they benefited from receiving chelation therapy. However, when asked to rate their satisfaction with their iron chelation therapy, patients receiving DFX were more likely to respond that they did not feel restricted in terms of their night activities. The differences in the means between the three groups were statistically significant (*P* = 0.0483). Furthermore, more patients receiving DFO responded that they were saddened by the unwanted side effects of their treatment (*P* = 0.0113) and that the treatment negatively influenced their body and skin appearance (*P* = 0.0276). 

**Figure 2:**
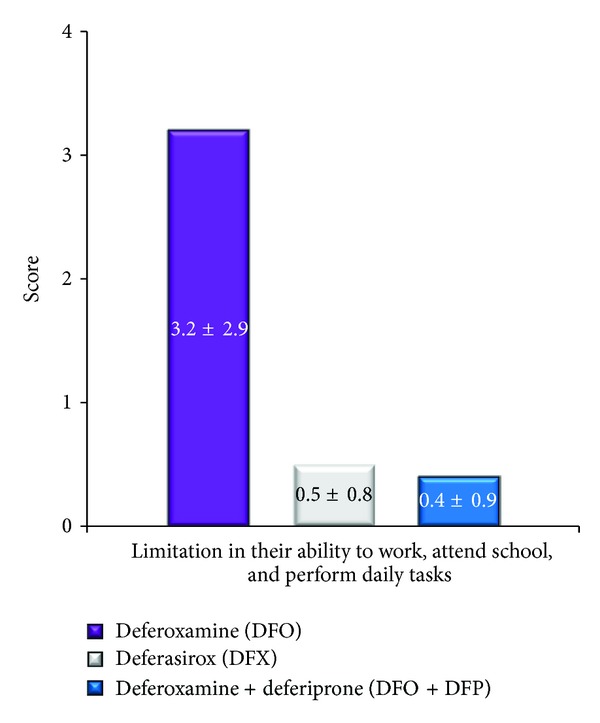
Effect of iron chelation therapy on the ability of the beta-thalassemia patients to perform their daily tasks. A statistically significant difference was noted among the three groups (*P* = 0.0066). Patients receiving DFO were more limited.

Furthermore, patients receiving DFO felt that their treatment limited their ability to work, to attend school, and to perform daily tasks (Figure 2). Specifically, the group receiving DFO were significantly more limited (i.e., had a higher score, 3.2 ± 2.9) than the DFX (0.5 ± 0.8) and DFO + DFP (0.4 ± 0.9) groups (*P* = 0.0066). 

**Figure 3:**
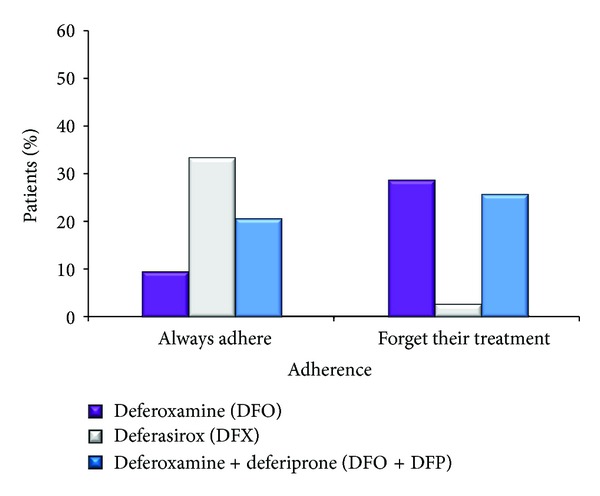
Adherence of patients to their treatment. The patients were asked to rate their adherence to treatment between five categories including the choices “always adhere,” “sometimes adhere” or “forget my treatment.” A statistically significant difference was noted among the three groups (*P* < 0.001). Adherence was the lowest among those receiving DFO.

The adherence to treatment rate was the lowest in the DFO group compared to the DFX group and the DFO + DFP group (*P* < 0.001) (Figure 3). In particular, 9.5% of those receiving DFO, compared to 33.3% of those receiving DFX and 20.5% of those receiving DFO + DFP, reported that they always adhered to their treatment, while the respective percentages of those that forgot to take their treatment were 28.6%, 2.7%, and 25.6% for the DFO, DFX, and DFO + DFP groups. 

**Figure 4:**
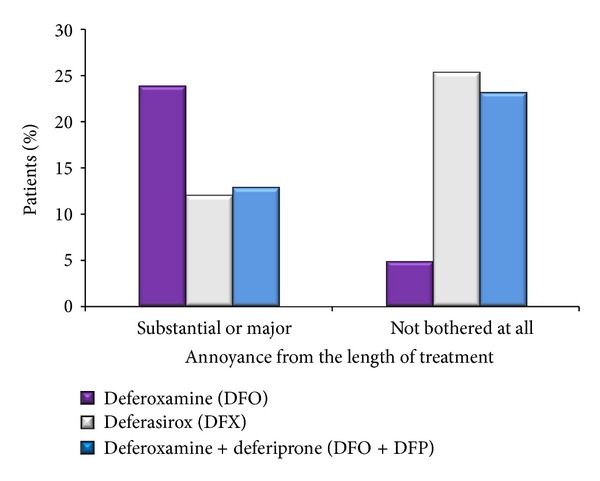
Annoyance from the length of treatment. Patients were asked to describe the extent of annoyance they felt from the length of their treatment. They could chose among 5 severity levels ranging from “major annoyance” to “none at all.” A statistically significant difference was noted among the three groups (*P* < 0.0293). Those receiving DFO reported that their treatment was hard or very hard to receive more often than patients of the other two groups.

**Figure 5:**
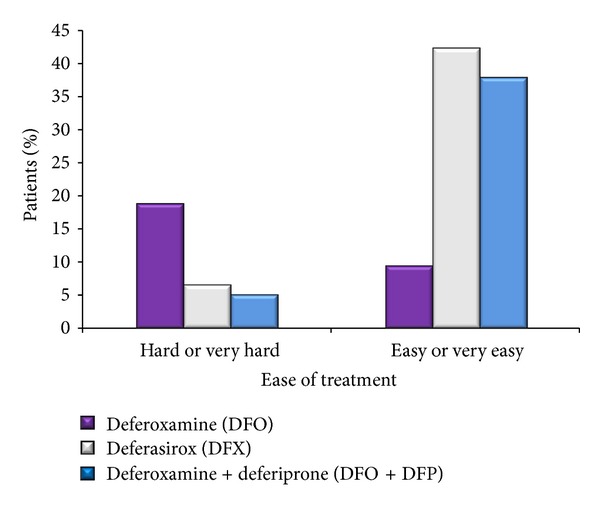
Ease of receiving treatment. Patients were asked to rate the difficulty of receiving their treatment on a 5-point scale ranging from very hard to very easy. A statistically significant difference was noted among the three groups (*P* = 0.0289). Those receiving DFO had the highest frequency of reporting that their the treatment was hard or very hard to receive and the lowest frequency of reporting that their treatment was easy or very easy to receive.

The percentages of patients that felt substantial or major annoyance from the length of their treatment were 23.8% for the DFO group, 12% for the DFX group and 12.8% for the DFO + DFP group. This is in sharp contrast to the percentages of 4.8, 25.3, and 23.1% of the DFO, DFX, and DFO + DFP groups of patients that were not bothered at all from the length of their treatment (overall *P* = 0.0293) (Figure 4). In addition, more patients receiving DFO (19%) felt that their treatment was hard or very hard to receive compared to 6.7% for DFX and 5.1% for DFO + DFP. This is in contrast to 9.5%, 42.7%, and 38.2% of those receiving DFO, DFX, and DFO + DFP, respectively, that felt that receiving their therapy was easy or very easy (overall *P* = 0.0289, resp.) (Figure 5). 

The three groups also differed significantly (*P* = 0.0316) in their satisfaction to their type of therapy (infusion, oral, or combination) with the majority of patients from all the groups preferring oral therapy. 

## 4. Discussion

One hundred and thirty-five adult beta-thalassemia transfusion-dependent homozygote patients took part in this study. The majority of the patients were single without children, in agreement with previous reports [12]. One-fifth of the patients were unemployed, a not very high percentage if one takes into account that the current unemployment rate of the general population in Greece is about 10% (and rising) and that a previous report showed that this rate was 16.2% two decades ago [13]. 

The DFO + DFP combination therapy offers a better control of serum ferritin levels, thus requiring less frequent DFO infusions [14]. It was thus not surprising that we found a decreased frequency of transfusions in the DFO + DFP combination group (*P* < 0.0001; Table 2). A higher percentage of DFO patients had comorbidities compared to the other two groups, except for thyroid disease, which was more prevalent in DFX patients. The presence of hepatic dysfunction in patients with homozygous beta-thalassemia has been correlated with iron overload in the liver as well as to chronic hepatitis [15, 16]. It is also notable that patients receiving DFX had the lowest prevalence of myocardiopathy which is in accordance with reports on the ability of DFX to prevent iron overload in the myocardium [17]. 

The highest rate of patient adherence to treatment was observed in the DFX patients. Adherence to therapy is the most important parameter for successful therapy. In fact low adherence of patients receiving DFO has been linked to the absence of clinical benefit [6]. In a previous study, low adherence to DFO was linked to smoking and to difficulties with self-administering the infusion [18]. 

Our results about satisfaction and ease of receiving their therapy matched those of previous studies, in which DFX was associated with increased satisfaction to treatment [19, 20]. Importantly, it was shown that switching chelators resulted in increased adherence, regardless of whether the patients switched from the oral to the intravenous chelator or vice versa, although the switch from DFO to DFP occurred more often [19]. 

According to previous studies, patients receiving DFO were more likely to suffer from depression, fatigue, dyspnea, and decreased physical functioning [21–23]. The majority of patients felt that they could participate in more activities if they were not receiving DFO [24] in accordance with the results of our study indicating that DFO limited the ability of patients to participate in sports and perform daily functions. Furthermore, the results of our study indicate that patients receiving DFO had lower self-esteem and worse PCS scores. These observations are in agreement with the results of the ITHACA study, in which the PCS score was low for patients receiving DFO [25] and with the study of Abetz et al., in which patients with DFO suffered from low self-esteem [21]. 

Of the specific components of the SF-36 questionnaire, the type of chelation treatment was proven to be statistically significantly associated with physical functioning, role limitations due to physical health problems, bodily pain, vitality, and mental health. Importantly, the results of our multivariate analysis indicate that the dependence of the PCS score on the type of chelation treatment was not confounded by anthropometric variables, such as gender, marital status, level of physical activity, presence of comorbidities, or smoking status. The importance of the SF-36 questionnaire and the results of the individual scales on the multidisciplinary actions that should be taken for patients with beta-thalassemia have been reported [26, 27]. 

The study is not without limitations. A randomized controlled clinical trial would be needed to ascertain the cause and effect relationship between quality of life and type of iron chelation therapy. Future studies should be of this design. However, its design as a prospective study of patients attending the hematology units of two major hospitals of Western Greece is ideal for reflecting daily clinical practice and the impact on the quality of life of these patients in “real-life.”

## 5. Conclusions

In conclusion, our study provides support for differences in the limitations of daily activities, physical activity, and quality of life among patients with transfusion-dependent beta-thalassemia depending on the type of their chelation therapy. Furthermore, the adherence to treatment, the ease and satisfaction from their therapy, and patient self-esteem differed along the three groups. This study highlights the importance of providing beta-thalassemia patients with the optimal chelation treatment based on their individual needs, in order to decrease the presence of unwanted comorbidities and to increase the quality of life, leading to increased adherence and thus resulting in optimal clinical benefit. Furthermore, our results highlight the need of the involvement of a multidisciplinary team in the management of patients with this disease.

## Figures and Tables

**Table 1:** Demographic characteristics and physical activity of the patients.

	DFO (*n* = 21)		DFX (*n* = 75)		DFO + DFP (*n* = 39)		
	*n*	%	*n*	%	*n*	%	*P* value
Gender							
Male	15	71.4	40	53.3	21	53.8	NS
Female	6	28.6	35	46.7	18	46.2	
Marital status							
Single	12	57.1	48	64.0	26	66.7	
Married	8	38.1	20	26.7	11	28.2	
Divorced	0	0.0	4	5.3	0	0.0	NS
Did not answer	1	4.8	3	4.0	2	5.1	
Parenthood							
Yes	5	23.8	12	16.0	10	25.6	
No	15	71.4	60	80.0	28	71.8	NS
Did not answer	1	4.8	3	4.0	1	2.6	
Physical activity							
None/low	8	38.1	17	22.7	3	7.7	
Moderate/high	13	61.9	54	72.0	36	92.3	NS
Did not answer	0	0.0	4	5.3	0	0.0	
Sports							
Yes	2	9.5	34	45.3	6	15.4	
No	19	90.5	36	48.0	33	84.6	<0.0001
Did not answer	0	0.0	5	6.7	0	0.0	
Smoking status							
Smoker	3	14.3	15	20.0	10	25.6	
Nonsmoker	6	28.6	36	48.0	10	25.6	NS
Did not answer	12	57.1	24	32.0	39	48.7	
Employment status							
Employed	16	76.2	60	80.0	28	71.8	
Unemployed	5	23.8	12	16.0	11	28.2	NS
Did not answer	0	0.0	3	4.0	0	0.0	

DFO: deferoxamine; DFX: deferasirox; DFP: deferiprone; NS: not significant. *P* values < 0.05 indicate statistical differences between the three groups.

**Table 2:** Disease characteristics.

	DFO	DFX	DFO + DFP	*P* value
Age of disease onset, years	2.1 ± 2.4	2.8 ± 4.5	2.3 ± 4.1	NS
Age at start of DFO treatment, years	13.1 ± 11.1	9.0 ± 9.6	11.1 ± 11.6	NS
Frequency of DFO therapy, times per week	5.2 ± 1.0	—	4.4 ± 1.9	<0.0001
Frequency of transfusions per month	2.2 ± 0.6	1.9 ± 0.5	2.1 ± 0.7	NS
Hemoglobin levels prior to transfusion, g/dL	9.5 ± 0.9	10.1 ± 3.4	9.7 ± 0.4	0.0208
Ferritin levels upon enrollment, ng/mL	1559.2 ± 1778.1	1738.0 ± 1636.9	1023.1 ± 944.3	NS

DFO: deferoxamine; DFX: deferasirox; DFP: deferiprone; NS: not significant. *P* values < 0.05 indicate statistical differences between the three groups.

All values are means ± SD.

**Table 3:** Frequency of comorbidities or prior splenectomy per group.

	DFO	DFX	DFO + DFP	*P* value
Myocardial dysfunction	33.3%	6.7%	15.4%	0.0058
Hepatic dysfunction	38.1%	6.7%	2.6%	<0.0001
Thyroid disease	28.6%	58.7%	53.8	0.0499
Hypogonadism	14.3%	10.7%	10.3%	NS
Splenectomy	71.4%	38.7%	48.7%	0.0319
Allergies	14.3%	9.3%	2.6%	0.0487

DFO: deferoxamine; DFX: deferasirox; DFP: deferiprone; NS: not significant. *P* values < 0.05 indicate statistical differences among the three groups.

**Table 4:** Association between SF-36 scales and chelation treatment.

SF-36 scale	Chelation treatment	*N*	Estimated mean score	95% CI for estimated mean score		*P* value*
	DFX	72	80.3	75.7	84.8	
Physical functioning	DFO + DFP	19	80.9	74.2	87.6	**0.048**
	DFO	33	68.4	59.6	77.3	

	DFX	71	79.9	71.5	88.3	
Role limitations due to physical health	DFO + DFP	17	76.5	64.2	88.8	**0.021**
	DFO	33	52.9	35.8	70.1	

	DFX	71	80.3	74.4	86.3	
Bodily pain	DFO + DFP	17	73.6	64.9	82.3	**0.015**
	DFO	33	60.7	48.6	72.8	

	DFX	70	51.6	47.4	55.9	
General health perceptions	DFO + DFP	17	53.1	46.9	59.3	0.111
	DFO	33	42.3	33.6	50.9	

	DFX	71	61.8	57.6	65.9	
Vitality	DFO + DFP	17	68.5	62.4	74.6	**<0.001**
	DFO	33	46.2	37.7	54.7	

	DFX	71	76.4	71.2	81.6	
Social functioning	DFOl + DFP	17	77.3	69.7	85.0	0.845
	DFO	33	73.5	62.9	84.1	

	DFX	71	77.9	69.6	86.3	
Role limitations due to emotional problems	DFO + DFP	17	71.4	59.2	83.6	0.338
	DFO	33	64.7	47.7	81.7	

	DFX	71	65.4	61.1	69.6	
Mental health	DFO + DFP	17	65.3	59.1	71.6	**0.001**
	DFO	33	46.8	38.1	55.5	

*Univariate analysis; DFO: deferoxamine; DFX: deferasirox: DFP: deferiprone; NS: not significant. *P* values < 0.05 indicate statistical differences among the three groups.
